# The Effect of Vitamin E on Oxidative Stress Indicated by Serum Malondialdehyde in Insulin-dependent *Type 2* Diabetes Mellitus Patients with Retinopathy

**DOI:** 10.2174/1874364101711010051

**Published:** 2017-03-31

**Authors:** Irini P. Chatziralli, George Theodossiadis, Prodromos Dimitriadis, Michail Charalambidis, Antonios Agorastos, Zisis Migkos, Nikolaos Platogiannis, Marilita M. Moschos, Panagiotis Theodossiadis, Petros Keryttopoulos

**Affiliations:** 1 2^nd^ Department of Ophthalmology, University of Athens, Athens, Greece; 2 Department of Internal Medicine, General Hospital of Veroia, Veroia, Greece; 3 1^st^ Department of Ophthalmology, University of Athens, Athens, Greece

**Keywords:** Oxidative stress, Diabetes mellitus, Malondialdehyde, Vitamin E

## Abstract

**Background:**

Several studies have focused on oxidative stress on diabetes mellitus (DM). Our purpose was to investigate the impact of oxidative stress on progression of diabetic retinopathy (DR) in insulin-dependent *type 2* DM patients, measuring serum malondialdehyde (MDA), as well as to examine the effect of vitamin E on DR progression in the above-mentioned patients.

**Methods:**

Participants in the study were 282 insulin-dependent *type 2* DM patients with DR. All participants underwent a thorough ophthalmological examination, so as to grade DR, along with serum MDA measurement. All participants received 300mg vitamin E daily for 3 months and were examined again. Serum MDA pre- and post-intake of Vitamin E was the main outcome.

**Results:**

Serum MDA was positively associated with DR stage, while there was a statistically significant difference pre- and post-intake of vitamin E in all DR stages. In a subgroup analysis of patients with proliferative DR, there was a significant difference at baseline between patients who have received prior laser photocoagulation and the treatment naïve patients, while after intake of vitamin E, no statistically significant difference was noticed.

**Conclusion:**

Oxidative stress has been found to play significant role in the pathogenesis and progression of DR, while vitamin E seems to reduce MDA levels and subsequent oxidative stress, suggesting that it might have protective role in DR progression.

## INTRODUCTION

Diabetes mellitus (DM) is associated with macro-vascular diseases and micro-vascular complications, such as neuropathy, nephropathy and retinopathy, induced by chronic hyperglycemia and subsequent hypoxia [1]. Diabetic retinopathy (DR) is one of the main causes of visual loss in individuals aged 20-64 years old and is present in almost all *type 1* DM patients, as well as in 60% of *type 2* DM patients 20 years after the onset of the disease [1-6]. Ηigh levels of glucose, causing apoptosis in vascular endothelial cells, and a great variety of hemodynamic changes i.e., increased blood viscosity, increased erythrocyte aggregation, alteration in erythrocyte permeability and increased adhesion of erythrocytes to endothelial cells, are considered to play an important role in the pathogenesis of DR [3, 6]. Furthermore, several candidate genes and gene polymorphisms have been implicated in the pathogenesis of DR [7, 8], while epigenetic mechanisms are thought to be also responsible for DR [9]. Specifically, in a recent study by Adardh *et al.*, DNA methylation was found to be a prospective marker of proliferative DR, predicting the development of DR [9].

Recent studies have focused on oxidative stress on DR, according to the “free radicals” hypothesis [10, 11]. Oxidative stress is described as the imbalance between excess production and/or impaired removal of reactive oxygen species (ROS) [12]. A variety of stimuli, such as hyperglycemia and hypoxia-hyperoxia, might increase the production of ROS at the retinal level, generating oxidative stress. Under these conditions a number of hyperoxides and aldehydes are produced, leading to microangiopathy in diabetes. Accumulation of ROS contributes not only to the pathogenesis of DR but also to the resistance of DR to reverse even after good glycemic control is achieved (metabolic memory phenomenon). Chronic oxidative stress causes damage in cell proteins, membrane lipids and nucleic acids [6, 12], disrupting cellular homeostasis. In order to compensate the effects of “free radicals”, there are defence mechanisms in the organism called anti-oxidants. The deficiency of anti-oxidant protection in DM patients increases the vulnerability to oxidative alterations of the retinal tissue and development of complications, as it may be the biochemical background for DM-associated endothelial dysfunction [9]. On the other hand, the potential effect of vitamin E, the major anti-oxidant in lipid phase, has been shown in DR by its free radicals scavenger activity outside the cell through non-enzymatic mechanisms [13, 14].

A sign of oxidative damage to cells and tissues is considered to be lipid peroxidation (LPO) [15]. Many years ago Sato *et al.* reported that plasma LPO levels of DM patients are higher than in normal population and LPO levels of DM patients with angiopathy are higher than in those without any complications [16], in accordance with other more recent studies [17-21]. Lipid peroxidation products are generated through oxidation of cell membrane lipids *via* ROS, being toxic to the microvascular cells, and degrade in a series of complexes to produce reactive carbon compounds, most commonly found malondialdehyde (MDA) [22, 23]. Pan *et al.* reported higher levels of MDA in patients with DM compared with controls and in patients with DR versus those without DR, also suggesting that oxidative stress may play a significant role in the development of DR [24]. As a result, levels of MDA are used as an index of LPO and in consequence of oxidative stress [5].

In light of the above, the purpose of our study was to investigate the impact of oxidative stress on progression of DR in insulin-dependent *type 2* DM patients, measuring serum MDA, an index of LPO. In addition, we examined the effect of vitamin E on DR progression in the above-mentioned patients.

## MATERIALS AND METHODOLOGY

Participants in our study were 282 insulin-dependent *type 2* DM patients with DR, 138 male and 144 female, with mean age 63.3±5.2 years (range: 50-70 years). The study was in accordance with the Tenets of Helsinki Declaration and was approved by the institutional review board. Written informed consent was obtained by all patients.

*Type 2* DM was diagnosed based on history, physical examination, laboratory investigations, and according to criteria laid down by the World Health Organization (WHO) [25]. All patients had relatively controlled DM with HbA1c <7.5% (58 mmol/ml). Patients, who were smokers or had history of alcohol use, cancer, coronary heart diseases, cerebrovascular diseases, liver diseases, chronic obstructive pulmonary disease, end-stage renal failure or diabetic nephropathy, use of antioxidant supplements and ocular surgery or intraocular inflammation, were excluded. All patients had body mass index (BMI) less than 30, without any specific diet. BMI was calculated by dividing the weight (kg) to the [length (m)]^2^. Female patients were all post-menopausal. All subjects were on oral anti-diabetics, statins and anti-hypertensives.

All participants underwent slit-lamp biomicroscopy and dilated fundoscopy. All participants presented DR, staged by an ophthalmologist, according to the Early Treatment Diabetic Retinopathy Study (ETDRS) criteria, as non-proliferative mild DR (Group I), moderate DR (Group II), severe DR (Group III) and proliferative DR (Group IV) [26]. Cases with media opacities, which prevented a detailed eye examination, were excluded from the study. Additionally, if diabetic macular edema was diagnosed, optical coherence tomography (Stratus OCT3, Carl Zeiss, Germany) was performed to confirm that and patients were excluded, so as to examine only those with DR.

Biochemical parameters of the participants who were chosen according to the criteria mentioned above were investigated at the Department of Biochemistry Laboratory. Approximately 10 ml of venous blood was taken from the antecubital vein following a 12 h fast. Five millilitres of the blood was transferred to tubes with heparin for the separation of erythrocytes, and 5 ml to plain tubes for the separation of serum. Plain tubes were kept at room temperature for about 30 min and then centrifuged for 15 min at 3000 rpm to separate the serum. Heparinised tubes were centrifuged for 10 min at 3000 rpm to separate plasma. Serum samples were immediately frozen and stored at -20° C for MDA assays. Serum MDA level was measured using Satoh’s method, based on the reactivity of thiobarbituric acid (TBA) [27]. Specifically, MDA creates a coloured complex, giving a maximum absorbance at 532 nm as it reacts with TBA. The MDA value was given in nanomoles per milliliter (nmol/ml).

All participants received 300mg vitamin E daily for 3 months and were examined again to measure MDA levels in each DR stage and in the whole sample at the 3-month follow-up.

For the description of patients’ characteristics, mean ± standard deviation (SD) was used for continuous variables and counts with percentages for categorical variables. Mann-Whitney-Wilcoxon test for independent samples (MWW for brevity) was performed to compare the baseline MDA levels between different groups of DR, while Wilcoxon matched-paired signed-rank test was used to make comparisons for the whole sample before and after vitamin E intake. Spearman’s correlation coefficients were used to calculate the association of DR with the DR stage. Statistical analysis was performed using SPSS 17.0 (SPSS Inc, Chicago, IL). Statistical significance was set at p<0.05.

## RESULTS

 Table **1** shows the demographic data of our sample.

Serum MDA was positively associated with DR stage (Spearman’s rho=+0.902, p<0.0001), as it is depicted in Fig. (**1**). Therefore, patients with proliferative DR (Group IV) had more increased MDA levels than those with non proliferative DR (Groups I,II,III), p<0.001, MWW test. At baseline, in proliferative DR subgroup, there was a statistically significant difference in MDA levels between patients who have received treatment with laser photocoagulation (Group IVa) and those who were treatment naïve (Group IVb), as it is shown in Fig. (**2**) (4.49 ± 0.12 for Group IVa *vs.* 4.87 ± 0.15 for Group IVb, p<0.0001, MWW).

**Fig. (2):**
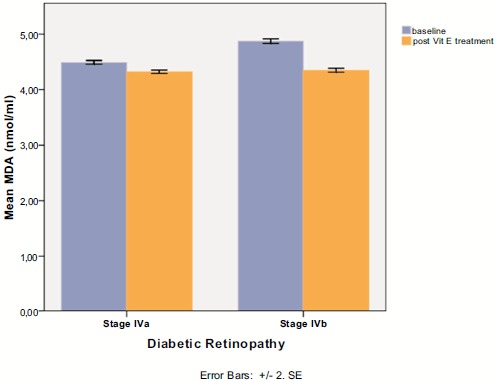
Sugroup analysis in stage IV diabetic retinopathy, between patients having received prior laser photocoagulation (IVa) and treatment naïve patients (IVb) before and after vitamin E intake.

There was a statistically significant difference pre- and post- intake of vitamin E in all DR stages (p<0.0001 in all stages, Wilcoxon matched-pairs signed-rank test), as it is shown in Table **2** and Fig. (**1**). In proliferative DR group, there was no statistically significant difference between the group having received laser treatment and the treatment naïve group, after the uptake of vitamin E (4.32 ± 0.11 for Group IVa *vs.* 4.35 ± 0.12 for Group IVb, p=0.240, MWW). However, there was a statistical significant difference in treatment response to vitamin E between the two subgroups of proliferative DR, as it indicated by MDA change (-0.17 ± 0.08 for group IVa vs -0.53 ± 0.12 for group IVb, p<0.001), clearly depicted in (Fig. **2**).

**Fig. (3):**
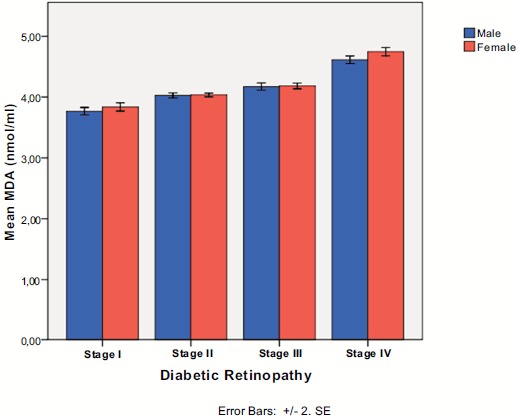
Serum malondialdehyde levels in each diabetic retinopathy stage according to gender.

Regarding gender, there was a borderline difference between males and females in serum MDA levels at baseline (4.16 ± 0.39 for males *vs.* 4.26 ± 0.41 for females for the whole sample, p=0.057, MWW test), as well as after intake of vitamin E (3.94 ± 0.35 for males *vs.* 4.01 ± 0.31 for females, p=0.051, MWW). In each stage separately, there was no statistically significant difference between males and females before and after vitamin E intake, as shown in (Fig. **3**).

## DISCUSSION

The principal message of our study is that serum MDA has been found to be significantly associated with the severity of DR in patients with *type 2* insulin-dependent DM. Additionally, vitamin E seems to reduce MDA levels and subsequent oxidative stress, suggesting that it might have a protective role in DR progression. As far as the gender is concerned, there was no significant difference in MDA levels between males and females before and after vitamin E intake.

Our findings are in line with previous studies, which have also found increased levels of oxidative stress markers in insulin-dependent DM patients and correlation of them with vascular complications of DM [28]. Increased LPO in DM has been associated with a variety of metabolic alterations; the most significant among them is hyperglycaemia, inducing the formation of thiose phosphate, whose oxidation causes overproduction of free radicals, leading to oxidative injury to blood cells, cross-linking of membrane lipids and proteins, increasing of cell aging, and vasoconstriction [29-31]. Therefore, the oxidative stress-mediated retinal neurodegeneration and the free radicals production, leading to progression of DR, may act in a common pathway to DM *per se* and to its complications [30-32].

In our study, MDA was more increased among PDR in comparison with NPDR patients. Marcino *et al.* reported that increased MDA is associated with oxidative stress and poor antioxidant defense, which promotes the progression of DR to its proliferative form [33]. This finding speculates that retinal microvascular complications is closely related to the severity of oxidative stress, as expressed as increased level of MDA among PDR patients. Indeed, the exact mechanism by which the oxidative stress contributes to diabetic complications remains unclear, but all biochemical alterations due to DM lead to anatomical and functional impairment in the retinal microvascular network, such as changes in blood flow in the retina, disruption of the blood-retina-barrier and consequently capillary occlusion and ischemia [34, 35]. Progressive reduction in capillary perfusion results in cotton-wool spots formation, venous beading, intraretinal microvascular abnormalities and neovascularization, as signs of DR and its progression to proliferative stage [35]. As a result, the early detection of hypoxia is very important of preventing potential severe retinal complications.

As far as vitamin E effect on oxidative stress is concerned, we found that serum MDA levels were reduced after vitamin E supplement intake along with hypoglycaemic drugs in all DR stages. Decreased activity of the antioxidant enzymes may increase diabetic patients’ susceptibility to oxidative damage [36, 37]. The potential benefit of vitamin E, the major antioxidant in lipid phase, has been shown in diabetic patients by its free radical scavenger activity outside the cell through non-enzymatic mechanisms [13, 14, 38]. It is worthy to note that vitamin E supplementation remains controversial, as some authors have found detrimental instead of beneficial effect due to prooxidant effect of vitamin E [39, 40]. However, prooxidant effect depends on doses of vitamin E, duration of intake and duration of DM [40]. Therefore, appropriate support for enhancing antioxidant supplies may help preventing complications of DM.

Another interesting finding of our study is that in patients with proliferative DR, there is a difference in response to vitamin E between patients having received laser photocoagulation treatment and treatment naïve patients, although at the 3-month follow-up the two subgroups did not differ significantly concerning MDA levels. This may suggest that in patients with previous laser photocoagulation, an alteration in the structure of the retina may take place, which modifies oxidative stress levels and subsequent treatment response. Furthermore, as MDA is a biomarker, it could be affected not only by oxidative stress but also by lifestyle factors, such as smoking and alcohol use, exercise and diet [41]. Thus, further studies are needed to reach a safe conclusion about the role of previous laser photocoagulation treatment in proliferative DR and its association with potential response in vitamin E intake.

Table (**2**). Serum malondialdehyde levels (nmol/ml) at baseline and 3 months after daily vitamin E intake in different diabetic retinopathy stages and for the whole sample.

All p values were derived using Wilcoxon matched-pairs signed-rank test.

A potential limitation of our study pertains to the fact that it lacks a control group. In addition, duration of DM was not recorded and it could be potentially associated with other findings of the study. However, the sample size is large and it is a prospective study, trying to scrutinize the role of vitamin E on progression of DR and oxidative stress, as it was expressed by MDA levels.

In conclusion, our results demonstrated that serum MDA levels were associated with DR stage, suggesting that oxidative stress play a significant role in DR progression. On the other hand, MDA levels were reduced in both non-proliferative and proliferative DR patients after vitamin E intake for 3 months. As a result, antioxidant supplementation, such as vitamin E, may be used as adjunctive treatment in patients with DR to reduce oxidative stress and to potentially protect from subsequent complications of DM.

## Figures and Tables

**Fig. (1):**
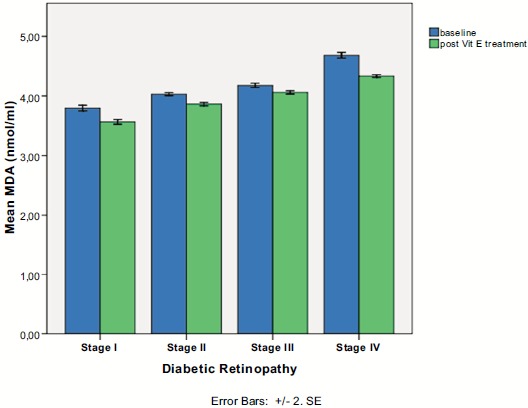
Serum malondialdehyde levels in each diabetic retinopathy stage pre- and post-intake of vitamin E.

**Table 1:** Baseline characteristics of our study sample.

	**Mean ± SD**
**Age (years)**	63.3 ± 5.2
**HbA1c (%, mmol/ml)**	6.3 ± 1.9 (45 ± 20.8)
**BMI**	26.6 ± 4.2
**MDA (nmol/ml)**	4.21 ± 0.4
	**N (%)**
**Gender** *Male* *Female*	138 (48.9) 144 (51.1)
**DR stage** *Stage I* *Stage II* *Stage III* *Stage IV*	74 (26.2) 65 (23.0) 49 (17.5) 94 (33.3)

BMI: body mass index; MDA: malondialdehyde; DR: diabetic retinopathy

**Table 2:** Serum malondialdehyde levels (nmol/ml) at baseline and 3 months after daily vitamin E intake in different diabetic retinopathy stages and for the whole sample.

	Baseline	Post vitamin E intake	*p* value
Stage I (n=74)	3.80 ± 0.20	3.57 ± 0.17	0.0001
Stage II (n=65)	4.03 ± 0.10	3.86 ± 0.12	0.0001
Stage III (n=49)	4.18 ± 0.13	4.06 ± 0.98	0.0001
Stage IV (n=94)	4.68 ± 0.23	4.34 ± 0.11	0.0001
All participants (n=282)	4.21 ± 0.4	3.98 ± 0.33	0.0001
